# Molecular Dissection of Symptom Determinants in Tomato Leaf Curl New Delhi Virus in Zucchini Through Mechanical Transmission

**DOI:** 10.3390/v17030294

**Published:** 2025-02-20

**Authors:** Thuy T. B. Vo, Eui-Joon Kil, Marjia Tabassum, Bupi Nattanong, Muhammad Amir Qureshi, Hyo-Jin Im, Giuseppe Parrella, Taek-Kyun Lee, Sukchan Lee

**Affiliations:** 1 Department of Integrative Biotechnology, Sungkyunkwan University, Suwon 16419, Republic of Korea; bichthuy251188@gmail.com (T.T.B.V.); marjia39@g.skku.edu (M.T.); gum.bupi@gmail.com (B.N.); amirq303@gmail.com (M.A.Q.); hyo990614@naver.com (H.-J.I.); 2 Faculty of Biotechnology, Ho Chi Minh City Open University, Ho Chi Minh City 700000, Vietnam; 3 Department of Plant Medicals, Andong National University, Andong 36729, Republic of Korea; viruskil@anu.ac.kr; 4 Institute for Sustainable Plant Protection of the National Research Council (IPSP-CNR), 80055 Portici, Italy; giuseppe.parrella@cnr.it; 5 Ecological Risk Research Department, Korea Institute of Ocean Science & Technology, Geoje 53201, Republic of Korea

**Keywords:** symptom variability, sap inoculation, chimeric infectious clones, pseudo-recombination

## Abstract

Among begomovirus species, tomato leaf curl New Delhi virus (ToLCNDV) is significant and stands out as a mechanically transmissible bipartite begomovirus originating from the Old World. However, the mechanisms underlying the mechanical transmission of different ToLCNDV strains remain understudied, as their natural transmission occurs via insect vectors. In this study, we investigated the mechanical transmissibility of two ToLCNDVs, one from Italy and another from Pakistan, in host plants. Several cucurbit species were screened, and symptom differences between the two ToLCNDV clones were observed only in zucchini when subjected to rubbing inoculation. The Italian isolate (ToLCNDV-ES) induced typical disease symptoms such as leaf curling, yellow mosaic, and internode stunting, whereas a normal phenotype was observed in zucchini mechanically infected with ToLCNDV-In (Pakistani isolate). Subsequently, a gene-swapping experiment between the two ToLCNDVs was conducted, and ToLCNDV-ES DNA-B was identified as a crucial factor in mechanical transmission. We then constructed chimeric mutant clones based on the DNA-B sequence and assessed their ability to induce symptoms in zucchini. These results indicated that the nuclear shuttle protein is a determinant of symptom development during ToLCNDV mechanical transmission. Moreover, several defense-related host genes showed significant changes in relative expression in different ToLCNDV clones, indicating their potential role in disease symptom development through the mechanical transmission of ToLCNDV. This is the first report comparing the mechanical transmissibility of two isolates of different ToLCNDV strains from the Mediterranean region and the Indian subcontinent in the same host plant, providing new insights into the virus’s pathogenicity across different geographic regions.

## 1. Introduction

Plant viruses cause significant economic losses in crops globally, affecting overall crop yield and quality [1]. The rising incidence of plant virus-related diseases highlights the need for in-depth research into their pathogenicity and molecular characterization, essential for preventing outbreaks. The transmission mode is one of the most important factors contributing to viral spread because plant viruses cannot penetrate plant cell walls. In addition to major transmission by insect vectors, mechanical transmission is significant as it represents an alternative pathway for virus spread leading to local lesions used for the identification, purification, and multiplication of plant viruses that develop on inoculated leaves [2]. According to over 3500 publications on plant virus species and their modes of transmission, 636 viruses were reported to be mechanically transmissible, whereas 114 viruses are not sap transmissible, accounting for 39.75% and 7.125% of the total 1600 plant viruses, respectively [3]. However, the rate of mechanical transmission was reported to be very low in the *Geminiviridae* family—the most devastating group of plant viruses, particularly in the prolific genus *Begomovirus* [4]. Approximately 20 of 445 recognized begomovirus species were reported to be mechanically transmissible to their natural hosts, with tomato leaf curl New Delhi virus (ToLCNDV) being a prominent member [5,6,7,8,9].

ToLCNDV is a crucial begomovirus with a genome consisting of two circular single-stranded DNA segments, designated as DNA-A and DNA-B. ToLCNDV has been recognized as a threat to cultivation in the Mediterranean region since 2012, following its first report in India in 1995 [10,11,12,13,14,15]. This virus can infect many economically important crops, making ToLCNDV a serious constraint for Solanaceae and Cucurbitaceae, including tomatoes, peppers, cucumbers, melons, zucchini, and pumpkins, particularly in Asia and the Mediterranean region. Like many phloem-limited viruses, ToLCNDV is transmitted by whiteflies (*Bemisia tabaci*) in nature. However, some studies showed that some ToLCNDV isolates can be transmitted via sap inoculation. The ToLCNDV isolated from Spain demonstrated mechanical transmissibility to the cucurbit germplasm, whereas other isolates from India (ToLCNDV-potato) and Taiwan (ToLCNDV-OM) could also infect host plants via sap inoculation [9,16,17]. Contrastingly, ToLCNDV-CB, isolated from a cucumber plant exhibiting symptoms, shares high genome sequence similarity with that of the ToLCNDV-OM isolate, although it is not mechanically transmissible [18]. The divergent modes of mechanical transmissibility among the ToLCNDV isolates may be due to the high genomic recombination and mutation rates of *Geminiviruses*; however, the underlying mechanism remains unclear [6]. Currently, only a few studies have explored and compared the mechanical transmissibility of different strains of ToLCNDV [19,20]. Furthermore, identifying transmission mechanisms is a critical step toward implementing effective control measures against this virus. Although no global agricultural impact of *Begomoviruses* via mechanical transmission was documented under natural field conditions, this transmission route cannot be dismissed entirely. Thus, understanding this mode of ToLCNDV transmission is essential, as it can contribute to viral proliferation through human activities, plant-to-plant contact, or other mechanical means. This aspect of virus behavior underscores the importance of studying host–virus interactions and the potential for mechanical transmissibility to improve management strategies for viral outbreaks, even though this mode was not reported in field conditions.

Here, we employed two ToLCNDV isolates for comparison: the Italian isolate Cum-45/16 (classified within subgroup I of the ToLCNDV-ES strain) and the Pakistani isolate BT20 (belonging to the ToLCNDV-In strain), which we reported earlier [13,21,22]. These two ToLCNDV isolates infect cucurbits via agro-inoculation, but mechanical transmission in zucchini showed differences: Italian ToLCNDV caused severe symptoms, while ToLCNDV isolated from Pakistan did not. Genetic swapping identified DNA-B as key to symptomatic transmission. Mutant clones with alterations in DNA-B ORFs and the intergenic region revealed nuclear shuttle protein (NSP) as a symptom determinant, with notable changes in defense-and NSP-related gene expression in asymptomatic versus symptomatic zucchini. To our knowledge, this is the first study to explore the mechanical transmissibility of two isolates from different ToLCNDV strains in zucchini. The results of this study contribute to our understanding of the differences in the pathogenic characteristics of the two ToLCNDV strains and raise awareness of their potential harm via mechanical transmission.

## 2. Materials and Methods

### 2.1. Plant Material and Virus Inoculum Preparation

Zucchini, melon, and pumpkin seeds were purchased from the NongWoo Bio Company (Seoul, Republic of Korea). The seeds were sown in soil, and the seedlings were kept in a growth chamber at 28/22 °C with 16/8 h light/dark periods after being transplanted into pots.

The viral inoculum was prepared via agro-inoculation using two infectious clones of ToLCNDV (ToLCNDV-ES and ToLCNDV-In from Italy (isolate Cum 45/16) and Pakistan (isolate BT20), respectively), which were obtained by constructing a partial tandem repeat of the full-length viral DNA, as described in our earlier studies [22]. Additionally, the sub-genomic components of the two ToLCNDV clones were swapped into two combinations, namely, ToLCNDV-India A + ToLCNDV-ES B (A_In_B_ES_) and ToLCNDV-ES A + ToLCNDV-India B (A_ES_B_In_), to create different inoculum combinations. Symptomatic leaves from the infected plants were used as the inoculum for the mechanical transmission experiment after confirmation by PCR.

### 2.2. Sap Inoculation of Two ToLCNDV Isolates

Symptomatic leaves from agro-infected plants were collected as inoculum for ToLCNDV sap inoculation experiments. Mechanical transmission was performed using COMAV buffer (50 mM potassium phosphate [pH 8.0], 1% polyvinylpyrrolidone 10, 1% polyethylene glycol 6000, 10 mM 2-mercaptoethanol, and 1% activated charcoal), following the method reported with some additions [17]. Briefly, leaf samples were ground in COMAV buffer at five ratios (leaf weight/buffer volume) of 1:1, 1:2, 1:3, 1:4, and 1:10. Healthy plant leaves were rubbed with carborundum powder, and the resultant sap was applied. Distilled water was used to wash the inoculated leaves for 10 min to remove the buffer and carborundum powder from the leaf surface. The inoculated plants were subsequently grown in a growth chamber, and virus detection was performed after 4 weeks.

For DNA-B quantification experiments, after sap inoculation from symptomatic zucchini leaves, the ToLCNDV-In DNA-B infectious clone was inoculated into plants using the pin-pricking method. *Agrobacterium* cells harboring DNA-B were cultured in 5 mL of LB broth medium containing kanamycin (50 µg/mL), rifampicin (50 µg/mL), and gentamycin (50 µg/mL) at 28 °C until the optical density at 600 nm (OD600) reached 1.0. The cells were then centrifuged, and the resulting pellets were resuspended in 5 mL of infiltration buffer (10 mM MgCl_2_, 10 mM MES, 200 µM acetosyringone). Following this, sap inoculation was performed in zucchini plants using symptomatic inoculum, and then 1 mL of cell suspension was used to introduce additional DNA-B into each plant by pinpricking the main apical shoot. Symptoms were observed, and PCR amplification was conducted at 28 days post inoculation (dpi).

### 2.3. Mutant Clone Construction

In this study, DNA-B was used to construct chimeric clones. Three chimeric sequences, comprising the intergenic region (IR) and two ORFs (BV1 and BC1) of ToLCNDV-In DNA-B on a ToLCNDV-India backbone (designated as ES_BIR_In, ES_BV1_In, and ES_BC1_In, respectively), were synthesized (Macrogen, Seoul, Republic of Korea) based on the sequences of two ToLCNDV isolates with tandem repeat constructs. The recombinant plasmid was obtained by ligating the pCAMBIA 1303 vector with the synthesized sequences digested with *Hind*III/*Spe*I. Similarly, a recombinant plasmid with the swapped sequence of ToLCNDV-In BV1 on the ToLCNDV-ES backbone (In_BV1_ES) was obtained. Recombinant plasmids were transformed into *Agrobacterium tumefaciens* strain GV3101 by freeze–thaw transformation. All chimeric clones were tested for infectivity in *N. benthamiana* and zucchini via agro-inoculation and mechanical transmission.

### 2.4. Virus Analysis by PCR and Quantitative PCR

The phenotype of zucchini plants was observed weekly after mechanical transmission. After 28 dpi, leaf samples were collected from each experiment, and genomic DNA was isolated using the FavorPrep Plant Genomic DNA Extraction Mini Kit (Favorgen, Ping-Tung, Taiwan) to detect ToLCNDV. PCR amplification was conducted using specific primer sets mentioned in the previous report [22].

Additionally, quantitative PCR (qPCR) was conducted on agro/sap-inoculated plants at 28 dpi to determine relative viral accumulation. Total DNA from the new leaves of the three plants was extracted and used as a template for qPCR as described in our earlier study [23]. The AC1 gene was used to check for DNA-A, and the BV1 gene was used to check for DNA-B.

To quantify the ratio between DNA-A and DNA-B of ToLCNDV under different infected conditions, we used pCAMBIA-1303 ToLCNDV-ES C1, ToLCNDV-ES BV1, ToLCNDV-In C1, and ToLCNDV-In BV1 as a standard following the absolute quantity standard curve (ES-C1: Y = −0.3279X + 12.024 and R2 = 0.993, ES-BV1: Y = −0.3273X + 11.137 and R2 = 0.986, In-C1: Y = −0.2951X + 11.698 and R2 = 0.994, In-BV1: Y = −0.3393X + 10.898 and R2 = 0.996) as described in a previous report [24]. The accumulation of ToLCNDV in infected zucchini was quantified by obtaining DNA copies in 1 μL and calculating the average DNA copy number (copy number/μL) per sample at 28 dpi. qPCR reactions were performed with specific primer sets (Table S1) based on the sequence of the V1 coding gene in DNA-A and BV1 coding gene in DNA-B of ToLCNDV using a Rotor Gene Q thermocycler (QIAGEN, Hilden, Germany), consisting of pre-denaturation at 95 °C for 5 min, followed by 40 cycles of denaturation at 95 °C for 10 min, annealing at 58 °C for 20 s, and extension at 72 °C for 20 s. Ubiquitin was used for internal normalization, and each reaction was replicated two times. Data and statistical analyses were conducted using the 2^−ΔΔCt^ method [25] and *t*-test in GraphPad Prism software 8.0.2 (GraphPad Software, Boston, MA, USA), respectively.

### 2.5. Quantitative Real-Time PCR of Host Genes

To assess the relative expression of host defense mechanisms under ToLCNDV-infected conditions, qRT-PCR was conducted using six genes including 26S proteasome subunit 6A homolog, pathogenesis-related protein, actin-related protein, Tornado 1, 4-Coumarate-CoA ligase-like protein, and NSP-interacting kinase 1 (NIK1) in zucchini at 28 dpi. These genes were selected based on previous reports of ToLCNDV resistance in cucurbit hosts such as melon, pumpkin, and cucumber, although no reports were available for zucchini [26,27,28]. For actin-related protein 4, the zucchini genome database did not accurately represent this protein. Therefore, the actin-related protein gene located on chromosome 8 was selected to assess its expression under ToLCNDV infection using the mechanical transmission method. Nucleotide sequences for each zucchini gene were obtained from the Cucurbit Genomics Database (CuGenDB) (http://cucurbitgenomics.org/) version 2. cDNA was synthesized from 1 µg of extracted total RNA using Oligo dT primers and CellScript mix as the template. qRT-PCR was performed using TB Green^®^ Premix Ex Taq™ II (Tli RNaseH Plus; TaKaRa Bio, Kusatsu, Japan), with 40 cycles—each cycle consisting of 10 s of denaturation at 95 °C, 15 s of annealing (with the annealing temperature selected according to the melting temperature of each primer), and 20 s of polymerization at 72 °C.

## 3. Results

### 3.1. Comparison of Mechanical Transmissibility of Two ToLCNDV Isolates in Cucurbit Species

Our earlier studies showed that two ToLCNDV clones could infect melons, zucchini, and pumpkins via agro-inoculation, inducing typical disease phenotypes. However, in this study, both clones demonstrated mechanical transmissibility in zucchini, although not in melon or pumpkin, as confirmed by PCR at 28 dpi when a 1:10 ratio of buffer to inoculum was used for sap inoculation. Various ratios (1:1, 1:2, 1:3, and 1:4) were tested to determine whether higher buffer/inoculum concentrations affected the mechanical transmissibility of the two ToLCNDV strains. The results indicated that zucchini was the only host adapted to the mechanical transmission of ToLCNDVs, regardless of the ratio used.

**Figure 1:**
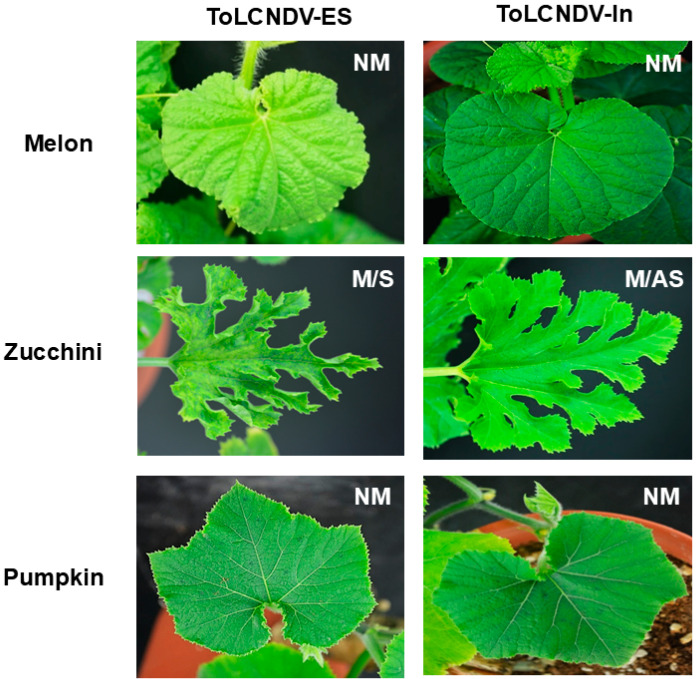
Mechanical transmissibility of two ToLCNDVs in cucurbit species. Symptomatic leaves from ToLCNDV-ES- and ToLCNDV-In-infected melon, zucchini, and pumpkin via agro-inoculation were used as inoculum to conduct mechanical transmission. The symptoms of inoculated plants were observed 2 weeks after sap inoculation. NM: non-mechanical transmissibility, M/S: mechanical transmission and induced symptoms, M/AS: mechanical transmission and no symptoms.

Additionally, differences in symptom development were observed in zucchini inoculated with the two ToLCNDV isolates (Figure 1). Plants infected with ToLCNDV-ES developed severe symptoms, including leaf curling, severe puckering, and yellowing of young leaves at 14 dpi, whereas no disease symptoms were observed in ToLCNDV-In-infected plants, even when maintained until 42 dpi.

### 3.2. Effect of ToLCNDV DNA-B on the Symptom Appearance Through Sap Inoculation in Zucchini

**Figure 2:**
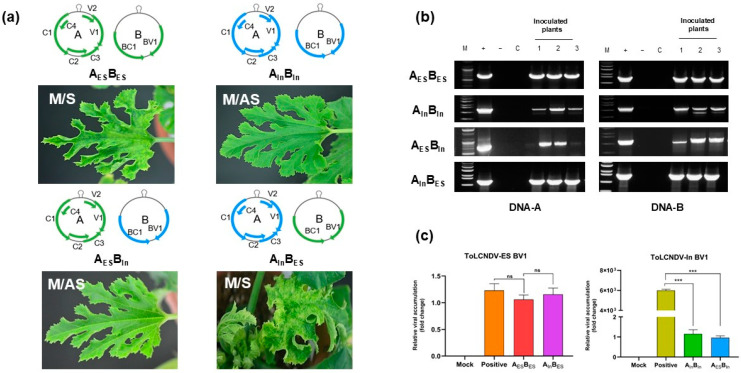
Effect of DNA B of ToLCNDV-ES on mechanical transmission in zucchini. DNA-A and DNA-B segments of two ToLCNDV were swapped and inoculated to zucchini plants by agro-inoculation. Then, the symptomatic leaves of each combination were used as inoculum to process sap inoculation on zucchini. (**a**) Phenotype of infected zucchini under different sub-genome combinations, M/S: mechanical transmission and induced symptom, M/AS: mechanical transmission and no disease phenotype. (**b**) PCR amplification results of inoculated plants, lane M: ladder (100 bp for A_ES_ and 1 kb for others), lane +: positive control, lane −: negative control, lane C: mock plants, lanes 1–3: inoculated plants. (**c**) Relative viral accumulations of two ToLCNDV BV1 genes on different infected plants, positive: agro-infected zucchini plants, A_ES_B_ES_: ToLCNDV-ES DNA-A + ToLCNDV-ES DNA-B, A_In_B_In_: ToLCNDV-In DNA-A + ToLCNDV-In DNA-B, A_ES_B_In_: ToLCNDV-ES DNA-A + ToLCNDV-In DNA-B, A_In_B_ES_: ToLCNDV-In DNA-A + ToLCNDV-ES DNA-B. The bar graph indicates the mean ± standard deviation (SD). The statistical comparison was performed with the unpaired *t*-test: *** *p* < 0.001, ns: not significant.

To determine the genomic region responsible for symptom development during the mechanical transmission of ToLCNDV in zucchini plants, four combinations were tested. These included wild-type combinations, ToLCNDV-ES-A/B (A_ES_B_ES_) and ToLCNDV-India-A/B (A_In_B_In_) as controls, and swapped genome combinations: A_ES_B_In_ and A_In_B_ES_. The sap inoculum was prepared from visibly symptomatic leaves of agro-inoculated zucchini plants using these four combinations. The data showed that ToLCNDV-ES DNA-B was required for symptom development following ToLCNDV infection via sap inoculation (Table 1, Figure 2). ToLCNDV-ES DNA-B, supplemented with either ToLCNDV-ES DNA-A or ToLCNDV-In DNA-A, induced disease symptoms at 14 dpi, whereas other combinations involving ToLCNDV-In DNA-B failed to induce symptoms in zucchini, although the viral genome was detected in all mechanically transmitted plants by PCR amplification (Figure 2a,b). The qPCR data showed that the relative ToLCNDV-In DNA-B accumulation in mechanically transmitted plants was significantly lower than that in the agro-infected plants. No difference in ToLCNDV-ES DNA-B levels was observed between the agro- and sap-inoculated zucchini (Figure 2c), indicating that ToLCNDV-ES DNA-B is essential for sap inoculation.

**Figure 3:**
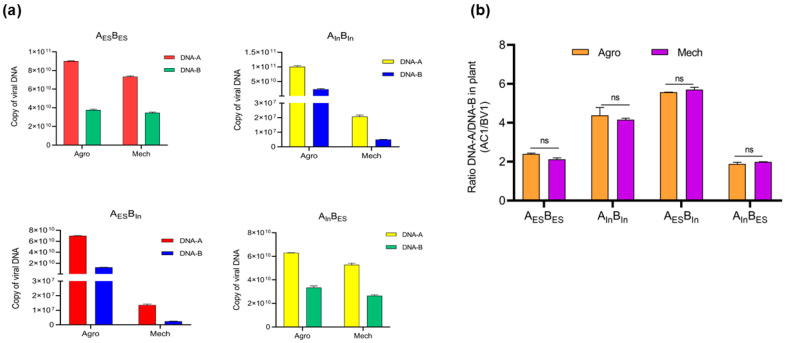
Quantities of ToLCNDV DNA-A and DNA-B under different agro/sap inoculations and their ratio in zucchini plants. Zucchini plants were inoculated with 4 different combinations (A_ES_B_ES_, A_In_B_In_, A_ES_B_In_, and A_In_B_ES)_ and sampled at 28 dpi. Quantitative viral DNA (expressed in copies/μL) using a standard curve of samples via agro-inoculation (Agro) and mechanical transmission (Mech) for DNA-A and DNA-B (**a**) and the DNA-A/DNA-B ratio (**b**). The AC1 and BV1 genes were used to check for DNA-A and DNA-B, respectively. Values are means ± SD, ns indicates not significant.

Bipartite begomoviruses such as ToLCNDV, DNA-A, and DNA-B are associated with and required for viral infection in plants. Some previous studies reported a constant ratio of the genomic components during transmission. Therefore, we evaluated the DNA-A/DNA-B ratio in all infected plants to confirm the effect of the DNA-B concentration on DNA-A or symptom development for each combination. The copy numbers of DNA-A in A_ES_B_ES_, A_In_B_In_, A_ES_B_In_, and A_In_B_ES_ correlated with DNA-B accumulation (Figure 3a). At 28 dpi, the DNA-A/DNA-B ratio via agro-inoculation was 2.395 ± 0.049, 4.385 ± 0.398, 5.57 ± 0.009, and 1.886 ± 0.082 in A_ES_B_ES_, A_In_B_In_, A_ES_B_In_, and A_In_B_ES_, respectively. Similarly, mechanically transmitted zucchini showed A/B ratios of 2.122 ± 0.077, 4.159 ± 0.08, 5.706 ± 0.118, and 1.993 ± 0.01 (Figure 3b, Table S2). These results indicated that the DNA-A/DNA-B ratio remained unchanged during agro- or sap- inoculation, even though the symptoms differed.

**Figure 4:**
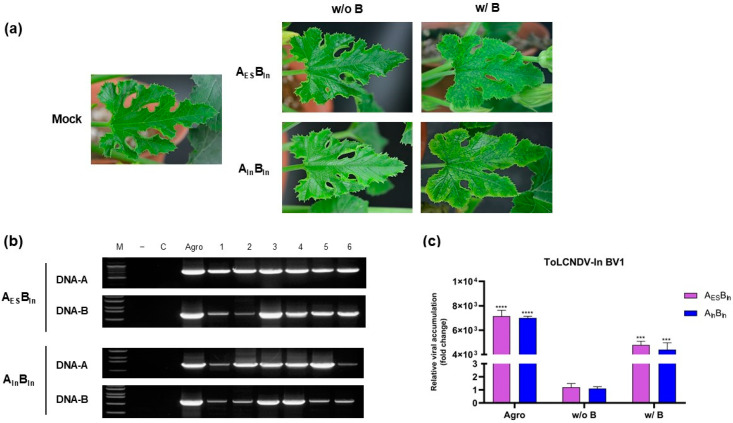
Infectivity of the additional DNA-B amount in mechanically transmitted zucchini plants. Sap-inoculated zucchini received additional application of 1 mL ToLCNDV-In DNA-B infectious clone and were observed for symptoms until 28 dpi. (**a**) The phenotype of inoculated plants under A_ES_B_In_ and A_In_B_ES_ infection, w/o B: only mechanical transmission, w/B: application of more DNA-B agro-infection clone after conducting mechanical transmission. (**b**) PCR analysis results after adding more DNA-B on 1% agarose gel, lane M: ladder (100 bp for A_ES_ and 1 kb for others), lane C: mock, lane Agro: infected samples via agro-inoculation, lanes 1–3: mechanically transmitted plants, lanes 4–6: mechanically transmitted plants with additional DNA-B infectious clone. (**c**) Relative DNA-B accumulation using qPCR. The data are presented as the mean ± standard deviation, **** *p* < 0.0001, *** *p* < 0.001.

Mechanically transmitted A_In_B_In_ and A_ES_B_In_ showed lower levels of DNA-B, which may be correlated with the appearance of symptoms in zucchini. To determine whether increasing the amount of DNA-B can induce disease symptoms during mechanical transmission, we increased DNA-B levels using infectious clones after sap inoculation. After four weeks, the results showed that yellow mosaic symptoms developed on plants only when additional DNA-B was introduced post-sap inoculation, whereas plants subjected to mechanical transmission alone showed no visible symptoms (Figure 4a). However, viral DNA was detected in all plants using PCR (Figure 4b). Relative DNA-B accumulation also correlated with symptom severity, as higher DNA-B levels produced more severe symptoms (Figure 4c). Plants that underwent mechanical transmission with the addition of DNA-B exhibited a less severe disease phenotype than those inoculated by agro-inoculation, with a lower accumulation of the virus. However, the accumulation in these plants was significantly higher than that in plants that did not show any symptoms following mechanical transmission alone. These data suggest that when the levels of ToLCNDV-In DNA-B are sufficiently high, the isolate is capable of inducing symptom development via mechanical transmission.

Conclusively, ToLCNDV-ES DNA-B plays a key role in symptom development during mechanical transmission. However, higher concentrations of ToLCNDV-In DNA-B can also induce disease symptoms, likely because of the differences between the DNA-B components of the two ToLCNDV isolates when expressed in plants following mechanical transmission.

### 3.3. Role of NSP in Different Symptom Adaptations of ToLCNDV Mechanical Transmissibility

**Figure 5:**
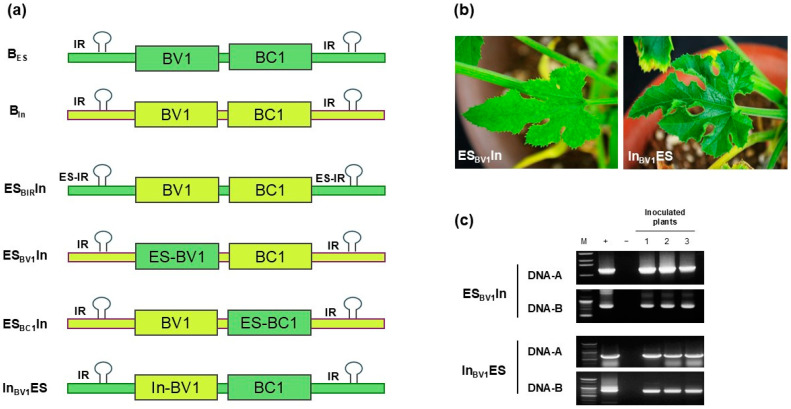
DNA-B chimeric clones in zucchini via mechanical transmission. Four chimeric clones were synthesized and transformed to agrobacteria to generate a series of DNA-B mutant clones. These clones include ES_BIR_In (IR of ToLCNDV-ES DNA-B in ToLCNDV-In backbone), ES_BV1_In (ToLCNDV-ES BV1 gene in ToLCNDV-In backbone), ES_BC1_In (ToLCNDV-ES BC1 gene in ToLCNDV-In backbone), and In_BV1_ES (ToLCNDV-In BV1 gene in ToLCNDV-ES backbone). (**a**) Genome map of different chimeric clones based on DNA-B sequence. (**b**) Phenotype of ES_BV1_In and In_BV1_ES at 28 dpi. (**c**) PCR results of inoculated zucchini. Lane M: ladder (1 kb for ES_BV1_In DNA-A and 100 bp for others), lane +: positive control, lane −: negative control, lanes 1–3: inoculated plants.

To identify the determinant gene(s) of ToLCNDV-ES DNA-B responsible for mechanical transmission, we synthesized three chimeric constructs by substituting the genomic regions containing BC1 (movement protein), BV1 (NSP), and IR of ToLCNDV-ES into the ToLCNDV-In DNA-B backbone (abbreviated as ES_BIR_In, ES_BV1_In, and ES_BC1_In) (Figure 5a). At four weeks post-sap inoculation, the chimeric construct ES_BV1_In induced moderate yellow mosaic symptoms, whereas the other constructs showed no visible symptoms in zucchini (Figure 5b, Table 1). Additionally, a chimeric clone harboring ToLCNDV-In BV1 in the ToLCNDV-ES backbone (In_BV1_ES) was constructed to confirm the role of NSP in the mechanical transmissibility of ToLCNDV. Viral accumulation in the inoculated plants was confirmed by PCR (Figure 5c). However, the infection rate of the mutant clones was lower than that of the wild-type ToLCNDV. These results suggest that NSP may be responsible for symptom induction during the mechanical transmission of ToLCNDV.

### 3.4. Expression of Related Host Defense Genes Under ToLCNDV Mechanical Transmission

Six host genes previously reported to be involved in ToLCNDV resistance were randomly selected to evaluate the role of the host defense system in response to ToLCNDV mechanical transmission (Table 2). This gene set included elements with diverse functions, such as the ubiquitin-proteasome system (26S proteasome subunit 6A), viral transport and replication (actin-related protein), cell expansion and vein formation (Tornado 1), the flavonoid pathway (4-Coumarate-CoA ligase), NSP-interacting kinase 1 (NSK 1), and pathogenesis-related protein. Homologs of these genes were selected based on the zucchini genome from CuGenDB, and their relative expression levels were measured.

**Figure 6:**
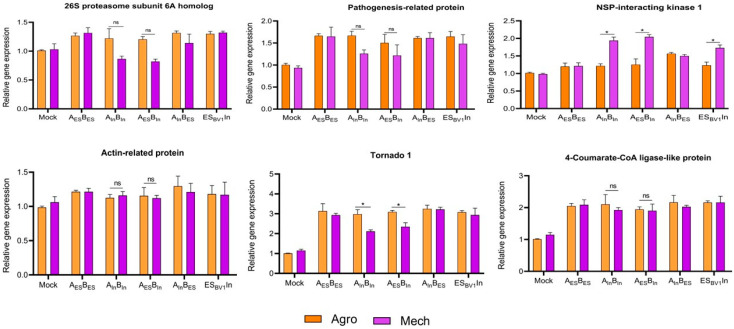
Relative expression levels of some host defense genes probably related to mechanical transmission in zucchini. The new leaves of mock and infected plants (wildtype: A_ES_B_ES_, A_In_B_In_; swapped genome: A_ES_B_In_, A_In_B_ES;_ and mutant clones: ES_BV1_In, In_BV1_ES) under agro- and sap-inoculated zucchini were collected at 28 dpi. qRT-PCR was conducted to check the relative expression of 6 genes including 26S proteasome, pathogenesis-related, actin−related, tornado 1, NSK 1, and 4−coumarate−CoA ligase in agro-inoculated plants (Agro), and sap-inoculated plants (Mech). The data are presented as the mean ± standard deviation, * *p* < 0.1, ns: not significant.

According to the qRT-PCR data, Tornado 1 and NSK 1 showed the largest differences in expression between zucchini infected via agrobacteria and sap inoculation, whereas no significant differences were observed in the other expressed genes (Figure 6, Table S3). The expression of 26S proteasome subunit 6A and actin-related protein was similar between healthy and infected plants (via agro/sap inoculation). 4-Coumarate-CoA ligase and pathogenesis-related gene were upregulated in infected plants compared to those in healthy controls, although no significant differences were observed between agro-infected and mechanically transmitted zucchini. Overall, Tornado 1 was highly upregulated in agro-infected symptomatic plants, while NSK 1 showed increased expression in mechanically transmitted plants with no visible symptoms. These initial data suggest that the expression of both genes may be altered during mechanical infection by ToLCNDV, although the underlying mechanism remains unclear.

## 4. Discussion

Mechanical transmission is a remarkable feature of ToLCNDV within the genus *Begomovirus*, which mostly comprises non-mechanically transmitted members [4,17]. This characteristic can make ToLCNDV more dangerous as mechanical transmission increases the potential for the virus to spread beyond a typical inset vector, especially in environments where physical contact between plants or human interaction with the infected material occurs. However, not all ToLCNDVs are capable of being mechanically transmitted to host plants. Here, we explored and compared the mechanical transmissibility of two previously identified cucurbit isolates of different ToLCNDV strains in Asia and the Mediterranean region in cucurbit species. Two ToLCNDV clones exhibited mechanical transmissibility only in zucchini, whereas other cucurbits (melon and pumpkin) were susceptible to agro-inoculation. This indicates that mechanical transmission varies across host species. Additionally, the differences in symptom appearance between Italian (ToLCNDV-ES strain) and Pakistani (ToLCNDV-In strain) isolates via sap inoculation in zucchini demonstrated the distinct adaptations of ToLCNDV strains from different geographic regions in the same host plants. These variations suggest that genetic factors or environmental influences could lead to differential interactions between the virus and host, influencing symptom development. Understanding the mechanisms underlying these differences could greatly aid research efforts toward developing resistance to this virus. To identify the genomic determinants responsible for the different zucchini phenotypes between the two isolates from the two ToLCNDV strains, various constructs of swapped clones were generated and introduced into the host plants via mechanical transmission. A genetic approach indicated that the DNA-B component of ToLCNDV-ES plays a critical role in symptom induction during mechanical transmission (Figure 2). Our results highlight the significant role of DNA-B in ToLCNDV mechanical transmission, which is consistent with that of an earlier report from a research group in Taiwan revealing that non-mechanical ToLCNDV became mechanically transmissible after swapping the DNA-B component with that of a mechanically transmissible ToLCNDV strain [20]. These data also coincide with findings for other begomoviruses, such as tomato yellow leaf curl Thailand virus and abutilon mosaic virus [4,8], suggesting that DNA-B is not only crucial for ToLCNDV but also plays a significant role in the mechanical transmission of other begomovirus species. The observed differences in symptom development between the two isolates may not only be attributed to variations in the viral genome but may also involve complex interactions between the virus and host plants. Host-specific responses to different viral strains may influence the severity and nature of symptoms, suggesting that isolate–host interactions could contribute to the differences observed in symptom expression.

Additionally, DNA-B accumulation appears to be related to symptom severity in infected plants and affects DNA-A accumulation [29]. In experiments involving sub-genome swapping, the relative accumulation of DNA-B differed significantly between the ToLCNDV strains, corresponding to the appearance of symptoms via rub inoculation. Specifically, ToLCNDV-ES DNA-B showed no change in accumulation throughout the experiments, regardless of whether it was agro- or sap-inoculated, and was consistently associated with severe disease symptoms. Contrastingly, ToLCNDV-In DNA-B showed high accumulation only in the agro-infected plants, whereas its relative accumulation was low in the sap-inoculated plants. Our data also showed a correlation between low DNA-A and DNA-B accumulation in asymptomatic plants, which may affect symptom induction in mechanically infected plants. A previous report on the temporal dynamics of DNA-A and DNA-B in the squash leaf curl China virus demonstrated the maintenance of a constant ratio between these two genomic components during infection and transmission [30]. Similarly, our findings indicate that to maintain this distinct ratio between DNA-A and DNA-B, the accumulation of DNA-A must remain low, with no significant difference between agro- and sap inoculation. This observation was consistent with that of our results. These findings suggest two possible explanations: (1) viral DNA-B itself may not amplify sufficiently to reach the necessary threshold for pathogenicity, which in turn affects DNA-A accumulation, limiting the ability of the virus to spread and cause disease symptoms; or (2) the defense system of the plant may be more effectively triggered by the ToLCNDV-In DNA-B component compared to that by ToLCNDV-ES, leading to more efficient suppression of the virus in asymptomatic plants, where viral DNA accumulation is significantly lower. To confirm the relationship between DNA-B levels and symptom induction, we conducted an experiment in which additional DNA-B was introduced into mechanically transmitted zucchini plants (Figure 4). The results showed that the addition of more ToLCNDV-In DNA-B after rub inoculation induced moderate symptoms, partially confirming our hypothesis that insufficient DNA-B levels are a potential factor in symptom development. The insufficient accumulation of DNA-B to induce the disease phenotype in zucchini may have resulted from differences in the genetics of the two ToLCNDV strains. Alternatively, this could be due to an unknown host plant response upon viral entry when the virus is transmitted directly through contact with infected leaves (mechanical transmission) or through an artificially constructed viral clone (agro-inoculation).

To investigate whether the differences in symptom development between the two ToLCNDV clones via mechanical transmission were due to differences in viral genes, we constructed several chimeric clones in which each of the ORFs of ToLCNDV-ES was exchanged with those of the ToLCNDV-In backbone. Like those of other begomoviruses, ToLCNDV DNA-B contains two genes (NSP and MP) and one intergenic region [31]. In some cases, MPs were reported to act as virulence factors in bipartite begomoviruses, contributing to symptom development [32,33], and our results from chimeric viral clones (ES_BIR_In, ES_BV1_In, and ES_BC1_In) suggest that the NSP in DNA-B is the key determinant required for symptom induction by different ToLCNDV isolates in zucchini via sap inoculation. Replacing the NSP of ToLCNDV-ES with that of the ToLCNDV-In backbone in ES_BV1_In clone led to a moderately yellow leaf mosaic phenotype. However, when ToLCNDV-ES lacked its own NSP and used an alternative ToLCNDV-In NSP, the In_BV1_ES clone did not produce disease symptoms in zucchini. These findings align with those of previous reports indicating that the NSP in DNA-B is a symptom determinant of ToLCNDV isolated from Pakistan in tobacco and tomatoes [34]. This study indicated that the NSP of ToLCNDV plays a significant role in activating plant defense responses, particularly the hypersensitive response, in species such as *Nicotiana tabacum* and tomato. Zhou et al. suggested that NSP contributes to avirulence in common beans, with specific amino acids in the N-terminus of NSP being the key determinant of this trait [35]. These findings highlight the dual role of NSP in triggering host defense responses, possibly contributing to broader plant health issues. Additionally, NSP plays a critical role in shuttling newly synthesized viral DNA from the nucleus to the cytoplasm. This function likely has a direct impact on the spread of the virus within the plant, potentially leading to insufficient viral accumulation in other tissues, which in turn may prevent the activation of pathogenicity and the visible induction of symptoms in the leaves. However, the precise mechanisms underlying these processes require further investigation. The amino acid differences in the NSP of the two ToLCNDVs may be a critical factor in explaining the observed differences in disease symptoms. Further research focusing on creating mutant clones of the virus to evaluate the roles of specific amino acids in NSP would be valuable. This type of study could clarify the mechanisms by which NSP contributes to symptom development and potentially identify the key factors mediating viral behavior during mechanical transmission.

While our experimental results focused on the differences between different ToLCNDVs in zucchini, the evidence regarding DNA-B and its role in symptom expression suggests broader implications for host–virus interactions. The observed differences in symptom severity linked to the presence of DNA-B and viral mutations support the idea that host factors influence the manifestation of the disease phenotype. To investigate the ToLCNDV–host interaction, we selected and examined the relative expression of four host genes reported to play a role in plant defense systems under ToLCNDV infection in different hosts [26,27]. These genes include (1) 26S proteasome, which is involved in restricting virus spread through the activation of the hypersensitive response and programmed cell death; (2) a pathogenesis-related gene—a key component of plant defense mechanisms; (3) an actin-related gene—implicated in viral transport and replication in other systems; and (4) Tornado 1, which is essential for cell expansion and vein formation. Our qRT-PCR results showed that only Tornado 1 expression was significantly upregulated in symptomatic plants (via agro-infection) compared to that in asymptomatic plants (via rub inoculation). This is similar to the observations made in tomatoes infected with ToLCNDV [36], which revealed that the elevation of Tornado 1 transcript levels during ToLCNDV infection may be related to the increased severity of symptoms. In our earlier studies on symptom development in cucumbers using NGS analysis, we found that certain genes in the flavonoid pathway may be involved in the symptom development process [28]. Based on the Cucurbit Genomics Database, we selected a homolog of the 4-Coumarate-CoA ligase-like protein in the zucchini genome and analyzed its expression following ToLCNDV inoculation. However, no significant differences were observed between agro- and sap-infected zucchini, suggesting that different hosts may adapt differently to ToLCNDV infection and that this gene may not be involved in the defense mechanisms of the host against ToLCNDV mechanical transmission. Some reports revealed that NSP acts as a pathogenicity determinant by interacting with and inhibiting NIKs, thereby suppressing antiviral responses and increasing plant susceptibility to geminivirus infections [37]. Therefore, we also selected this gene homolog in the zucchini genome to examine its expression under different inoculation methods and found a significant upregulation in asymptomatic plants. This supports the role of NIK 1 in the mechanical transmission of ToLCNDV, as NSP is recognized as a symptom determinant. Overall, the differential expression of certain host genes between agro-inoculated (symptomatic) and sap-inoculated (normal) plants, particularly those related to NSP, partially explains the differences in the adaptation between a whole virus naturally infecting a plant and an artificial viral clone introduced into the plant. However, further studies are needed to identify more host genes and explore virus–host interactions using different transmission methods. The observed alterations in the expression of host genes in response to ToLCNDV infection suggest a potential link between gene regulation and virus–host interactions during mechanical transmission. The upregulation or downregulation of specific defense-related genes may play a critical role in modulating host defense mechanisms, thereby affecting the ability of the virus to replicate and spread. Further investigations into potential gene expression profiles, in combination with other host–virus interaction studies, are necessary to elucidate their broader implications for virus transmission dynamics and plant resistance.

## 5. Conclusions

**Figure 7:**
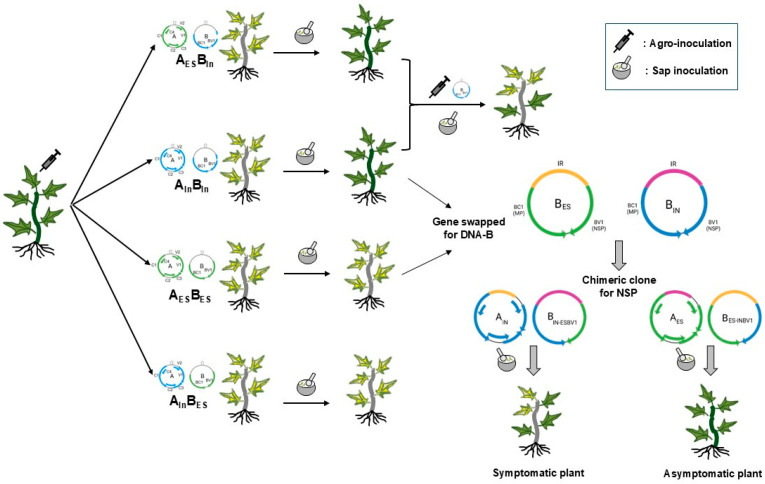
Overview of mechanical transmission of two ToLCNDV clones in zucchini in this study. This figure provides a comprehensive summary of all experiments conducted to investigate the mechanical transmissibility of ToLCNDV. It also consolidates key findings to facilitate a clearer understanding of the steps involved and the outcomes observed in our study.

Conclusively, our study highlights the mechanical transmissibility of ToLCNDV and compares the transmission rates of two ToLCNDV isolates representing Asian and Mediterranean strains. Figure 7 summarizes the methodologies and key findings supporting the mechanical transmission of ToLCNDV in zucchini, offering a clear overview of the spread of this virus through non-natural transmission modes, although the exact underlying mechanisms remain unclear. Although no significant global impact of mechanical transmission of begomoviruses was reported under natural field conditions, our findings underscore the potential risk that mechanical transmission could pose to the spread of ToLCNDV, especially given the differences observed in symptom development. These results are a necessary step toward raising awareness of the possible role of mechanical transmission in viral outbreaks even in the absence of clear evidence from natural conditions.

## Figures and Tables

**Table 1:** Summary of infectivity of all clones used in this study in zucchini by mechanical transmission. The inoculated plants were observed every week, and infectivity was confirmed by PCR amplification at 28 dpi.

Experiment	IC	Agro-Inoculation		Mechanical Transmission	
		Infectivity	Symptom	Infectivity	Symptom
Subgenome swapped	A_ES_B_ES_	9/9	Severe leaf curl, yellow mosaic, stunting	11/12	Severe leaf curl, yellow mosaic, stunting
	A_In_B_In_	8/9	Severe leaf curl, yellow mosaic, stunting	8/12	No symptoms
	A_ES_B_In_	8/9	Severe leaf curl, yellow mosaic, stunting	8/12	No symptoms
	A_In_B_ES_	9/9	Severe leaf curl, yellow mosaic, stunting	10/12	Severe leaf curl, yellow mosaic, stunting
Chimeric	ES_BIR_In	6/9	Leaf curl, mosaic, mild stunting	5/9	No symptoms
	ES_BV1_In	7/9	Leaf curl, mosaic, mild stunting	4/9	Mild mosaic
	ES_BC1_In	7/9	Leaf curl, mosaic, mild stunting	6/9	No symptoms
	In_BV1_ES	7/9	Leaf curl, mosaic, mild stunting	5/9	No symptoms

**Table 2:** Primers used in RT-PCR for amplifying defense-related candidate genes in zucchini. Six candidates were chosen based on previous studies, and relative expressions were checked under different ToLCNDV transmissions.

Target Gene	Primer Sequence (5′-3′)	Target Size	Gene Location
26S proteasome subunit 6A homolog	F: AACCCTGAAGATGACGCAGA	100 bp	*chr11*: 7268831 .. 7273953 (+)
	R: TAGTCTGGCGAGTGGATGTC		
Pathogenesis-related protein	F: TGTTTCGTTCCTCTCTCGCT	166 bp	*chr15*: 5346921 .. 5347412 (+)
	R: GAGTGCACCAATCGACAGTC		
Actin–related protein	F: ATCATCAGACTTGGCCACCA	126 bp	*chr 8*: 2026365 .. 2036546 (−)
	R: CTACAGCAACAGCATCGACC		
Tornado 1	F: TCTGCAGCAACCAACAACTC	197 bp	*chr2*: 5147191 .. 5151428 (−)
	R: TCACAGCTTCCACAAATGCC		
4-Coumarate-CoA ligase-like protein	F: TCTGCAGCAACCAACAACTC	181 bp	*chr9*: 4145298 .. 4149751 (−)
	R: TCACAGCTTCCACAAATGCC		
NSP-interacting kinase 1 (NIK1)	F: CATTTGCTCATCTGGGTCGG	195 bp	*chr9*: 1462093 .. 1466184 (−)
	R: CGTTGCCTCCACCAAATGAA		

## Data Availability

The original contributions of this study are included in the article. Further inquiries can be directed to the corresponding author.

## Supplementary Materials

The following supporting information can be downloaded at: https://www.mdpi.com/article/10.3390/v17030294/s1, Table S1: Primers used for viral analysis via PCR and qPCR in this study; Table S2: Viral copy number (copies/µL) under different ToLCNDV infections via agro-inoculation and mechanical transmission. Table S3. Ct values of qRT-PCR to check the relative expression of several host genes in zucchini. The values are presented as mean ± SD.
