# Supplementary material for: Molecular Dissection of Symptom Determinants in Tomato Leaf Curl New Delhi Virus in Zucchini Through Mechanical Transmission
Source: Viruses. 2025 Feb 20;17(3):294. doi: 10.3390/v17030294 (PMC11946352; doi:10.3390/v17030294)
Supplement: Supplementary file 1 [file viruses-17-00294-s001.zip › viruses-3470843-supplementary.pdf]

Table S1. Primers used for viral analysis via PCR and qPCR in this study.

Purpose	Primer	Sequence (5'-3')	Target size (bp)
Detection (PCR) of chimeric clones	ES-A	F: AAGCTTAAAACGTGTCGTTTCGATCTGG<br>R: CTCGAGTAACATCACTAACACAC	725
	ES-BV1	F: ATGGCTTTTCCTTCTCCATA<br>R: TTATCCAATGTAATTCAGAA	807
	In-A	F: AAGCTTCCATAAAACTTGTCGTTTCGATC<br>R: GGTACCTAAATATGCTTGGTGTA	1336
	In-BV1	F: ATGGCTTTTCCTTCTCCTTA<br>R: TCATCCAATGTAATTAAGAA	807
Quantitative (qPCR)	ES-qC1	F: GGGGATCAACGTCGTCAATG<br>R: GGGATAGTAGGACGGGCAAA	135
	ES-qBV1	F: TCCATATTCCACTCCTCGCC<br>R: GGATCGCCAAACAATTCGGT	159
	In-qC1	F: ATAGCTGCTGTTCGGACCTT<br>R: TGATGACGTTGATCCCCACT	154
	In-qBV1	F: AAACACAGGAGGGATCGGAG<br>R: ATCATGTCAGATGTGCGCAC	169

Table S2. Viral copy number (copies/µl) under different ToLCNDV infections via agro-inoculation and mechanical transmission.

Genotype	Agro-inoculation			Mechanical transmission		
	Copies of viral DNA (mean ± SD)		Ratio A/B	Copies of viral DNA (mean ± SD)		Ratio A/B
	DNA-A	DNA-B		DNA-A	DNA-B	
$A_{ES}B_{ES}$	$9.01 \pm 0.03 \times 10^{10}$	$3.76 \pm 0.09 \times 10^{10}$	2.395 ± 0.049	$7.34 \pm 0.08 \times 10^{10}$	$3.46 \pm 0.08 \times 10^{10}$	2.122 ± 0.077
$A_{In}B_{In}$	$10.05 \pm 0.35 \times 10^{10}$	$2.3 \pm 0.29 \times 10^{10}$	4.385 ± 0.398	$2.08 \pm 0.9 \times 10^{7}$	$5 \pm 1.4 \times 10^{6}$	4.159 ± 0.08
$A_{ES}B_{In}$	$6.99 \pm 0.02 \times 10^{10}$	$1.25 \pm 0.07 \times 10^{10}$	5.57 ± 0.009	$1.36 \pm 0.5 \times 10^{7}$	$2.3 \pm 0.14 \times 10^{7}$	5.706 ± 0.118
$A_{In}B_{ES}$	$6.29 \pm 0.07 \times 10^{10}$	$3.34 \pm 0.14 \times 10^{10}$	1.886 ± 0.082	$5.29 \pm 0.12 \times 10^{10}$	$2.65 \pm 0.07 \times 10^{10}$	1.993 ± 0.01

Table S3. Ct values of qRT-PCR to check the relative expression of several host genes in zucchini. The values are presented as mean ± SD.

Gene	Agro-inoculation (Agro)						Sap inoculation (Mech)					
	Mock	$A_{ES}B_{ES}$	$A_{In}B_{In}$	$A_{ES}B_{In}$	$A_{In}B_{ES}$	$ES_{BV1}In$	Mock	$A_{ES}B_{ES}$	$A_{In}B_{In}$	$A_{ES}B_{In}$	$A_{In}B_{ES}$	$ES_{BV1}In$
26S proteasome subunit 6A homolog	1.013 ± 0.014	1.265 ± 0.049	1.22 ± 0.17	1.205 ± 0.049	1.315 ± 0.035	1.3 ± 0.042	1.03 ± 0.099	1.315 ± 0.092	0.865 ± 0.049	0.82 ± 0.042	1.14 ± 0.156	1.32 ± 0.028
Pathogenesis-related protein	1.005 ± 0.035	1.665 ± 0.049	1.670 ± 0.099	1.505 ± 0.191	1.615 ± 0.035	1.65 ± 0.113	0.935 ± 0.049	1.65 ± 0.212	1.26 ± 0.085	1.22 ± 0.24	1.615 ± 0.12	1.485 ± 0.205
NSP-interacting kinase 1 (NIK1)	1.015 ± 0.021	1.2 ± 0.099	1.217 ± 0.061	1.255 ± 0.163	1.565 ± 0.035	1.235 ± 0.092	0.985 ± 0.021	1.215 ± 0.092	1.94 ± 0.099	2.045 ± 0.064	1.5 ± 0.042	1.73 ± 0.085
Actin-related protein	0.985 ± 0.021	1.215 ± 0.021	1.125 ± 0.049	1.155 ± 0.12	1.295 ± 0.148	1.18 ± 0.127	1.06 ± 0.085	1.215 ± 0.049	1.16 ± 0.057	1.12 ± 0.042	1.21 ± 0.127	1.17 ± 0.184
Tornado 1	1.01 ± 0.014	3.135 ± 0.375	2.98 ± 0.226	3.095 ± 0.078	3.25 ± 0.184	3.080 ± 0.085	1.15 ± 0.071	2.935 ± 0.092	2.115 ± 0.078	2.347 ± 0.207	3.22 ± 0.113	2.94 ± 0.339
4-Coumarate-CoA ligase-like protein	1.02 ± 0.014	2.055 ± 0.078	2.105 ± 0.304	1.945 ± 0.078	2.165 ± 0.219	2.166 ± 0.057	1.17 ± 0.07	2.090 ± 0.156	1.925 ± 0.078	1.905 ± 0.205	2.025 ± 0.049	2.16 ± 0.198
